# Self-disorders in schizophrenia-spectrum disorders: a 5-year follow-up study

**DOI:** 10.1007/s00406-017-0837-3

**Published:** 2017-09-01

**Authors:** Julie Nordgaard, Lars Siersbæk Nilsson, Ditte Sæbye, Josef Parnas

**Affiliations:** 1 Early Psychosis Intervention Center Region Zealand, University Hospital Copenhagen, Smedegade 10, 4000 Roskilde, Denmark; 2 Mental Health Center Glostrup, University Hospital Copenhagen, Broendbyoestervej 160, 2605 Broendy, Denmark; 3 Department of Clinical Epidemiology, Frederiksberg Hospital, Nordre Fasanvej 57-59, 2000 Frederiksberg, Denmark; 4 Center for Subjectivity Research, University of Copenhagen, Karen Blixens Plads 8, Copenhagen S, 2300 Copenhagen, Denmark

**Keywords:** Self-disorders, GAF, Temporal, Persistence, Schizophrenia-spectrum

## Abstract

Self-disorders have been hypothesized to be an underlying and trait-like core feature of schizophrenia-spectrum disorders and a certain degree of temporal stability of self-disorders would therefore be expected. The aim of the study was to examine the persistence of self-disorders measured by the Examination of Anomalous Self Experiences over a time span of 5 years. 48 patients with schizophrenia-spectrum disorders were thoroughly assessed for psychopathology at baseline and 5 years later. Self-disorders were assessed by the Examination of Anomalous Self Experiences. The level of self-disorders was same at the two occasions for the full Examination of Anomalous Self Disorders and for four out of the five domains. For one domain, the level of self-disorders increased slightly from baseline to follow-up. The correlations between baseline and follow-up were moderate. 9 out of the 13 most-frequently rated items at baseline showed equal frequencies at follow-up. The baseline level of self-disorders predicted global symptomatic, but not functional outcome. Self-disorders measured by the Examination of Anomalous Self Experiences show a high level of temporal persistence over 5 years and predict symptomatic outcome.

## Background

The first reports in contemporary psychiatry pointing to a disorder of the self appeared at the turn of the millennium [18, 25]. The main idea from these studies was that a disorder of the self is the core feature of schizophrenia-spectrum disorders. It was not a novel insight, but can be found in nearly all foundational texts on schizophrenia [1, 2, 10, 14, 17, 31].

It is postulated that the affected structure is the ‘minimal’ or ‘core’ self, which enables us to experience ourselves as self-same subjects who experience the world from our own first-person perspective [3, 6]. It means that we have an immediate, but tacit sense of bodily self-presence, and all our mental states are imbued with a sense of subjectivity. In schizophrenia-spectrum disorders this sense-of-self becomes weakened, often leading to hyperreflectivity and a feeling of being ephemeral, not fully existing. One’s field of experience (e.g., thoughts or sensations) may be felt as increasingly distant and spatialized (e.g., thoughts are experienced as physical objects) [30].

In 2005, a scale (Examination of Anomalous Self Experiences—EASE) targeting a range of anomalous self-experiences indicative of the basic self-disorder was published [26]. Since the publication, several research groups have used this scale to explore anomalies of self-experience in schizophrenia-spectrum disorders, ultra high-risk populations, unselected help-seeking adolescents, and the general population [8, 13, 19, 21, 27, 29].

We have previously reported temporal persistence of self-disorders measured by an early precursor of the EASE [20]. However, no reports of the persistence of EASE-measured abnormalities have yet been published. This issue is of theoretical and clinical importance; if we consider the underlying self-disorder as a core trait feature of the schizophrenia-spectrum, then we would expect some degree of stability of the abnormal phenomena.

### Aim of the study

In this study, we examined EASE-measured self-disorders (SD) in a sample of first-admission patients with schizophrenia-spectrum disorders at admission and at a follow-up 5 years later.

## Methods

### Sample

The original sample comprised 100 consecutive first-admission patients of a psychiatric facility in Copenhagen, for details see [21].

To be included in the study, the patients had to be considered capable of tolerating lengthy interviews, because one of the study goals was to examine the adequacy/efficacy of different psycho-diagnostic interview approaches [22]. This requirement naturally excluded aggressive, agitated, and/or severely psychotic patients. Additional exclusion criteria comprised primary or clinically dominating alcohol/substance abuse, history of brain injury, mental retardation, organic brain disorder, and age >65 years. Due to ethical concerns, involuntarily admitted and forensic patients were also excluded. This selection procedure resulted in a sample of comparatively “mildly” ill patients, yet still requiring hospital admission.

The follow-up took place 5 years later. We wished to re-examine the patients who at the index evaluation received a ICD-10 schizophrenia-spectrum disorder diagnosis, i.e., a diagnosis of non-affective, non-organic psychosis, or schizotypal disorder.

The patients participated on the condition of informed consent and a relevant Medical Ethical Committee approved the study.

### Interviews and assessments

The details of the diagnostic assessments at baseline are published elsewhere [22, 23]. Briefly, at baseline all patients were interviewed with the SCID-I and the Schizotypal Personality Disorder module from the SCID-II [5], the OPCRIT-scale (an extract of the PSE) [16], expanded with additional items from the SADS-L [4], the EASE [26], perceptual items from the BSABS [7], a checklist of the First Rank Symptom continua [12], and a Mental Status Examination [10, 11, 15]. Additionally, the patients were assessed with respect to life history, overall psychosocial functioning, family history of mental disorder, and the evolution of psychopathology.

The baseline timeframe for the assessment of self-disorders were lifetime and at follow-up self-disorders were assessed within the last 18 months.

Finally, all patients were allocated a ‘Best-Estimate Consensus Life-Time’ ICD-10 diagnosis and a ‘Best-Estimate Consensus Life-Time’ DSM-IV diagnosis by JP and JN, who jointly reviewed all available, diagnostically relevant information.

74 patients were diagnosed within the schizophrenia-spectrum according to the ICD-10, 68 of these also fulfilled a DSM-IV diagnosis within the schizophrenia-spectrum.

At follow-up, the psychopathological assessment was identical to the baseline battery described above, except for the SCID being excluded. The interviewer at follow-up was blind with respect to detailed psychopathological information from the index interviews.

The interviewer at follow-up (LSN) also allocated each participant a lifetime ICD-10 and a lifetime DSM-5 diagnosis. The interviewer was a clinical psychiatrist who had been trained in the application of the EASE instrument. More specifically, the inter-rater reliability between the baseline and follow-up interviewers was tested on 18 patients resulting in a Cohen’s kappa of 0.81.

In accordance with previous publications, we looked only for the presence or absence (not severity or duration) of the EASE items and explored the latter as dimensions (i.e., summing up the items rated as present, we only used main items). We scored 0 for absent or questionably present and 1 for present. We only included the main items and not sub-types in the analyses. The EASE shows a high degree of internal consistency (Chronbach’s alpha 0.903) [21].

Since we did not use the Positive and Negative Syndrome Scale (PANSS) in the baseline assessment to obtain these measures, we constructed proxy scales for positive and negative symptoms by adding nonoverlapping items selected from the interview schedule, for details see [21]. This procedure was applied to both the baseline and the follow-up data.

### Sample attrition

We only succeeded to obtain full personal interviews with 48 subjects of the original group of 74 (65%). 12 declined to participate in the follow-up study, 12 were lost to follow-up, 1 emigrated, and 1 was in forensic treatment.

### Data analysis

In all analyses, we used the ICD-10 diagnoses.

We tested for potential differences in the self-disorder scores between baseline and follow-up using mixed models and ANOVA adjusting for domain as a repeated variable. Because of questionable normal distribution of residuals tested by Shapiro–Wilks test, we also compared baseline and follow-up for the total EASE scores and for each of the five domains with the non-parametric Wilcoxon signed-rank test.

The correlations between baseline and follow-up were tested with Spearman’s rho, two-tailed.

We tested if baseline EASE score predicted GAF follow-up by univariate linear regression.

Furthermore, we compared the baseline-remainders and the baseline-dropouts with Wilcoxon signed-rank test. We used Fischer’s exact test to test for independence between the variable for remainders/dropouts and (1) gender; (2) educational level; (3) marital status; and (4) diagnosis. We used the McNemar to test the independence between the variable for the baseline/follow-up and variable (2)–(4).

We used SPSS version 24, Stata version 14.1, and SAS version 9.4.

## Results

Table 1 shows the differences for the following variables at baseline and follow-up, and for the 26 patients included at baseline, but not at follow-up (dropouts): gender; marital status; the diagnostic groups (non-affective psychosis or schizotypal personality disorder); educational level; age; self-disorders; Global Assessment of Functioning-Symptom scale (GAF-S); Global Assessment of Functioning-Function scale (GAF-F); and the positive and negative symptoms scales. The Wilcoxon signed-rank test results show that the mean for both GAF-S and GAF-F increased significantly (*P* < 0.0001) from baseline to follow-up, reflecting at better level of functioning and a lower level of symptoms at follow-up.

**Table 1:** Sample profiles and EASE scores at baseline and at 5-years follow-up

	Baseline remainers (BA)	Follow-up (FO)	Dropouts (DO)	Difference FO-BA	BA vs FO	BA vs DO
Total *N*	48	48	26			
Gender	*N* (%)	*N* (%)	*N* (%)	(Kappa)		*P* value. Test for independence
Male	18 (37.5)	18 (37.5)	8 (30.8)	(1.0)		0.62
Female	30 (62.5)	30 (62.5)	18 (69.2)			
Social status	*N* (%)	*N* (%)	*N* (%)	Mc Nemar^a^ (Kappa)	*P* value^b^	*P* value. Test for independence
Married	18 (37.5)	26 (54.2)	12 (46.2)	8 (0.67)	0.008**	0.62
Alone	30 (62.5)	22 (45.8)	14 (53.8)			
Diagnosis				Mc Nemar^a^ (Kappa)	*P* value^b^	*P* value. Test for independence
Non-affective psychosis	30 (62.5)	35 (73.0)	15 (57.7)	2.8 (0.58)	0.18	0.80
Schizotypy	18 (37.5)	13 (27.0)	11 (42.3)			
Highest level of education	*N* (%)	*N* (%)	*N* (%)	Bowker’s^d^ (Kappa)		
Primary school or less	17 (35.4)	10 (20.8)	16 (61.5)	10.3 (0.61)	0.41	NS^c^
High school	18 (37.5)	25 (52.1)	5 (19.2)			
College	5 (10.4)	6 (12.5)	1 (3.9)			
Started university	4 (8.3)	1 (2.1)	3 (11.5)			
Finished university	4 (8.3)	6 (12.5)	1 (3.9)			

	Mean (sd)	Mean (sd)	Mean (sd)	Mean (sd)	*P* value. Wilcoxon’s signed rank test for equal means	
Age in years, mean	25.5 (6.33)	30.1 (6.38)	26.2 (8.13)	4.6 (0.71)	<0.0001***	0.79
Self-disorders						
Total EASE score	20.63 (8.14)	21.92 (7.21)	15.96 (6.30)	1.29 (7.28)	0.40	0.03*
Domain 1	7.42 (3.18)	7.44 (3.01)	6.31 (2.74)	0.02 (3.23)	0.85	0.15
Domain 2	7.71 (3.28)	8.73 (2.33)	5.88 (3.04)	1.02 (2.97)	0.03*	0.04*
Domain 3	2.1 (1.46)	2.1 (1.67)	1.69 (1.46)	0 (1.82)	0.95	0.21
Domain 4	0.96 (0.99)	1.21 (0.92)	0.50 (0.65)	0.25 (0.98)	0.09	0.06
Domain 5	2.44 (2.06)	2.44 (1.77)	1.58 (1.30)	0 (1.65)	0.81	0.16
Positive and negative symptoms scales						
Positive symptoms	4.33 (3.72)	4.00 (3.96)	5.11 (4.65)	−0.33 (4.35)	0.69	0.75
Negative symptoms	3.60 (1.83)	5.04 (1.13)	3.15 (1.67)	1.44 (1.84)	<0.0001***	0.27
GAF	*N* = 47	*N* = 47	*N* = 26			
GAF-S, mean (*N* = 47)	37.21 (8.08)	49.87 (9.53)	39.04 (8.41)	12.66 (10.21)	<0.0001***	0.31
GAF-F, mean (*N* = 47)	42.4 (10.82)	56.13 (11.32)	44.23 (9.71)	13.72 (11.05)	<0.0001***	0.37

We re-analyzed the data using the DSM-IV. Although the sample size changes the results are basically the same

*SD* standard deviation, *GAF-S* global assessment of functioning-symptoms, *GAF-F* global assessment of functioning-function. Significance level = 0.05

^a^McNemar test statistic = (*b* − *c*)^2^/(*b* + *c*)

^b^ *P* value from the exact binomial test evaluating the McNemar test statistic

^c^Non-Significant, but Chi Square test is not valid due to expected count <5 in many cells

^d^Test of symmetry 49/7 + 1/3 + 1/1 + 4/2

Five patients (10.4%) changed diagnosis from schizotypal disorder to schizophrenia at the follow-up assessment.

Several of the patients completed further education and got married during the 5 years.

*P* values from the mixed analysis (0.13) and the ANOVA (0.13) adjusting for domain as repeated variable showed that the mean SD score can be assumed to be identical at the both occasions when we adjust for domain. Additionally, we tested the full EASE scale and each of the five domains independently of each other (due to questionably normal distributed residuals). The mean differences between SD at baseline and follow-up are also presented in Table 1. For the full EASE scale and for four of the five domains (domain 1, 3, 4, and 5), it can be assumed that the mean SD score is the same at baseline and at follow-up. For domain 2, the mean score slightly increased at follow-up.

The 26 dropouts had a statistically significant lower level of SD at baseline than the 48 remainders.

Table 2 shows the correlation matrix for the EASE scale, the five EASE domains, and the GAF scores. We found a moderate (*ρ* = 0.484, *P* < 0.01) correlation between baseline and follow-up of the full EASE. Additionally, the correlation between baseline SD and baseline GAF-S was moderate (*ρ* = −0.378, *P* < 0.01) and so was the correlation between baseline SD and follow-up GAF-S (*ρ* = −0.382, *P* < 0.01).

**Table 2:** Correlations between the full EASE scale and the five domains at baseline and follow-up

		EASE		Domain 1		Domain 2		Domain 3		Domain 4		Domain 5	
		BA	FO	BA	FO	BA	FO	BA	FO	BA	FO	BA	FO
EASE	BA	–											
	FO	0.479**	–										
Domain 1	BA	0.706**	0.409**	–									
	FO	0.290*	0.811**	0.439**	–								
Domain 2	BA	0.862**	0.385**	0.436**	0.201	–							
	FO	0.538**	0.841**	0.367*	0.588**	0.464**	–						
Domain 3	BA	0.627**	0.416**	0.183	0.18	0.568**	0.494**	–					
	FO	0.231	0.715**	0.261	0.449*	0.195	0.501**	0.331*	–				
Domain 4	BA	0.504**	0.330*	0.129	0.146	0.526**	0.341*	0.310*	0.043	–			
	FO	0.291*	0.504**	0.207	0.269	0.224	0.340*	0.057	0.238	0.450**	–		
Domain 5	BA	0.716**	0.397**	0.309*	0.155	0.566**	0.443**	0.438**	0.122	0.370**	0.290*	–	
	FO	0.475**	0.658**	0.203	0.337*	0.355*	0.452**	0.380**	0.435**	0.349*	0.456**	0.661**	–

*BA* baseline, *FO* follow-up. Spearman two-tailed

** *P* < 0.01; * *P* < 0.05

Correlations between the full EASE, and the positive and negative symptoms scales were not significant except for the correlation between positive symptoms and EASE at follow-up (*ρ* = 0.530, *P* < 0.01). There was no temporal change in the level of positive symptoms and a slight increase in the level of negative symptoms.

In Table 3, the 13 most-frequently rated items at baseline are displayed. Additionally, the table shows the *P* value from the McNemar test (*P* values below 0.05 is in bold, indicating a significant difference between baseline and follow-up) and the frequencies of the items’ presence at baseline and at follow-up. Equal proportions at baseline and follow-up were found for 9 out of the 13 items.

**Table 3:** McNemar’s test for equal frequencies at baseline and follow-up at item level, for the 13 most-frequently rated items at baseline

EASE item	McNemar2-sid exact *P* ^a^	Frequency at baseline	Frequency at follow-up	McNemar test statistic^b^
Thought pressure (item 1.3)	0.3438	0.9	0.81	1.6
Anxiety (item 2.13)	0.625	0.88	0.92	1
Ruminations–obsessions (item 1.6)	0.125	0.85	0.96	3.57
Hypohedonia (item 2.17)	0.0574	0.85	0.69	4.57
Hyperreflectivity (item 2.6)	0.002	0.75	0.96	10
Ambivalence (item 1.9)	1	0.69	0.71	0.08
Diminished initiative (item 2.16)	0.2668	0.69	0.79	1.92
Diminished sense of basic self (item 2.1)	0.0129	0.67	0.88	7.14
Perceptualisation of inner thought (item 1.7)	0.5811	0.65	0.71	0.69
Derealization (item 2.5)	0.0013	0.65	0.35	10.89
Primary self-reference (item 5.1)	1	0.65	0.65	0
Cenestesia (item 3.7)	0.0007	0.58	0.27	11.84
Disorder of short-term memory (item 1.13)	0.5034	0.58	0.5	0.8

Bold indicates non-equal frequencies between the two times

^a^ *P* value from the exact binomial test

^b^McNemar test statistic = (*b* − c)^2^/(*b* + *c*)

Finally, baseline SD affected follow-up GAF-S [slope estimate −0.346 (95% CI 0.62; −0.07), *P* = 0.017]; thus, pointing to higher levels of SD at the initial assessment predicting higher symptomatic levels at follow-up (in this analysis, we omitted one subject who exhibited an extraordinary outlier value which could not be adapted to the analytic model). There was no effect of baseline SD on GAF-F at the follow-up.

## Discussion and conclusion

A limitation of this study is that we only succeeded to obtain follow-up interviews with 65% of the patients, but this figure corresponds to most follow-up studies. However, there were no differences with respect to standard global psychopathological (positive and negative symptoms measures) or demographic characteristics between the participants and the dropouts.

To the best of our knowledge, this is the first report on the persistence of SD measured by the EASE across 5 years in a sample of schizophrenia-spectrum patients. The total level of SD at the two occasions was not statistically different. Looking at the domains individually there was a minor increase for domain 2, measuring self-awareness and presence. This little increase may perhaps be ascribed to methodological reasons. This domain of experiences is difficult to verbalize, and the patients may have been made more aware of these phenomena and thus were better able to verbalize it at the second interview.

Baseline SD correlated with GAF score both synchronically and across occasions. Apart from the expected intercorrelations within the EASE and the correlation between EASE and positive symptoms scale at follow-up, there were no other significant correlations. We have previously shown correlations between EASE, and positive symptoms scale and negative symptoms scale, respectively; however, the current sample is considerably smaller and diagnostically more homogeneous [21].

Notably, SD predicted global symptomatic, but not functional outcome. The functional and symptomatic levels at follow-up may reflect that all patients were hospitalized at baseline, but not at follow-up.

With respect to singular items, 9 of the 13 most-frequently rated items at baseline had equal frequencies at follow-up.

This study demonstrates a temporal persistence of self-disorders in schizophrenia-spectrum patients. This persistence is consistent with the view of SD as a fundamental feature of schizophrenic psychopathology, as advocated by the founders of the concept [2].

We have previously reported from another sample that was assessed for SD using a pre-EASE scale, and showed a similar pattern of results. The pre-EASE scale is not directly comparable to the EASE, because it was a post hoc scale constructed due to our evolving interest in SD. The reader may compare our Table 3 with Table 1 from [20]. The pre-EASE scale comprises less than half of the items corresponding to the EASE items. Of the 13 most frequent items rated in this study five items were also included in the pre-EASE scale. All these five items were also among the most frequently rated items in the previous study. All five pre-EASE items were among the nine most-frequent rated items at both initial and follow-up assessment [20].

We have elsewhere proposed that SD constitute a phenomenological core of the schizophrenia-spectrum [24, 30].

It must be emphasized that although all patients were psychiatric in-patients at the time of the first assessment (first admission), the patient sample is relatively mild at a symptomatic level, see [28].

## References

[1] Berze J (1914). Die primäre insuffizienz der psychischen aktivität: Ihr wesen, ihre erscheinungen und ihre bedeutung als grundstörungen der dementia praecox und der hypophrenen überhaupt.

[2] Bleuler E (1911). Dementia praecox oder gruppe der schizophrenia.

[3] Damasio A (2010). Self comes to mind: constructing the conscious brain.

[4] Endicott J, Spitzer RL (1978). A diagnostic interview: the schedule for affective disorders and schizophrenia. Arch Gen Psychiatry.

[5] First M, Gibbon MSW, Spitzer RL, Williams JBW (2002). User's guide for SCID-I structured clinical interview for DSM-IV-TR axis I disorders Research version.

[6] Gallagher S, Zahavi D (2008). The phenomenological mind: an introduction to philosophy of mind and cognitive science.

[7] Gross G, Huber G, Klosterkötter J, Linz M (1987). Bonner skala für die beurteilung von basissymptomen.

[8] Haug E, Lien L, Raballo A, Bratlien U, Oie M, Andreassen OA, Melle I, Moller P (2012). Selective aggregation of self-disorders in first-treatment dsm-iv schizophrenia spectrum disorders. J Nerv Ment Dis.

[9] Jaspers K (1963) General psychopathology (tr. Hoenig, j & hamilton, m). The John Hopkins University Press, London

[10] Kety SSRD, Wender PH, Schulsinger F, Jacobsen B (1975). Mental illness in the biological and adoptive families of adopted individuals who have become schizophrenic: a preliminary report based on psychiatric interviews. Genetic research in psychiatry.

[11] Kety SSRD, Wender PH, Schulsinger F (1968). The types and prevalence of mental illness in the biological and adoptive families of adopted schizophrenics. The transmission of schizophrenia.

[12] Koehler K (1979). First rank symptoms of schizophrenia: questions concerning clinical boundaries. Br J Psychiatry.

[13] Koren D, Reznik N, Adres M, Scheyer R, Apter A, Steinberg T, Parnas J (2013). Disturbances of basic self and prodromal symptoms among non-psychotic help-seeking adolescents. Psychol Med.

[14] Kraepelin E (1919). Dementia praecox und paraphrenie.

[15] Matthysse S, Holzman PS, Gusella JF, Levy DL, Harte CB, Jorgensen A, Moller L, Parnas J (2004). Linkage of eye movement dysfunction to chromosome 6p in schizophrenia: additional evidence. Am J Med Genet Part B Neuropsychiatr Genet.

[16] McGuffin P, Farmer A, Harvey I (1991). A polydiagnostic application of operational criteria in studies of psychotic illness. Development and reliability of the opcrit system. Arch Gen Psychiatry.

[17] Minkowski E (1927). La schizophrénie.

[18] Moller P, Husby R (2000). The initial prodrome in schizophrenia: searching for naturalistic core dimensions of experience and behavior. Schizophr Bull.

[19] Nelson B, Thompson A, Yung AR (2012). Basic self-disturbance predicts psychosis onset in the ultra high risk for psychosis “prodromal” population. Schizophr Bull.

[20] Nordgaard J, Handest P, Vollmer-Larsen A, Saebye D, Pedersen JT, Parnas J (2017). Temporal persistence of anomalous self-experience: a 5 years follow-up. Schizophr Res.

[21] Nordgaard J, Parnas J (2014). Self-disorders and the schizophrenia spectrum: a study of 100 first hospital admissions. Schizophr Bull.

[22] Nordgaard J, Revsbech R, Saebye D, Parnas J (2012). Assessing the diagnostic validity of a structured psychiatric interview in a first-admission hospital sample. World Psychiatry: Off J World Psychiatr Assoc.

[23] Nordgaard J, Sass LA, Parnas J (2013). The psychiatric interview: validity, structure, and subjectivity. Eur Arch Psychiatry Clin Neurosci.

[24] Parnas J, Henriksen MG (2016). Mysticism and schizophrenia: a phenomenological exploration of the structure of consciousness in the schizophrenia spectrum disorders. Conscious Cogn.

[25] Parnas J, Jansson L, Sass LA, Handest P (1998). Self-experience in the prodromal phases of schizophrenia: a pilot study of first admissions. Neurol Psychiatry Brain Res.

[26] Parnas J, Moller P, Kircher T, Thalbitzer J, Jansson L, Handest P, Zahavi D (2005). Ease: examination of anomalous self-experience. Psychopathology.

[27] Raballo A, Pappagallo E, Dell’ Erba A, Lo Cascio N, Patane M, Gebhardt E, Boldrini T, Terzariol L, Angelone M, Trisolini A, Girardi P, Fiori Nastro P (2016). Self-disorders and clinical high risk for psychosis: an empirical study in help-seeking youth attending community mental health facilities. Schizophr Bull.

[28] Raballo A, Parnas J (2012). Examination of anomalous self-experience: initial study of the structure of self-disorders in schizophrenia spectrum. J Nerv Ment Dis.

[29] Raballo A, Saebye D, Parnas J (2011). Looking at the schizophrenia spectrum through the prism of self-disorders: an empirical study. Schizophr Bull.

[30] Sass LA, Parnas J (2003). Schizophrenia, consciousness, and the self. Schizophr Bull.

[31] Schneider K (1959). Clinical psychopathology.

